# On data benchmarking and verification of discrete granular simulations

**DOI:** 10.1016/j.dib.2024.110252

**Published:** 2024-02-27

**Authors:** Jose Salomon, Catherine O'Sullivan, Fernando Patino-Ramirez

**Affiliations:** Department of Civil and Environmental Engineering, Imperial College London, London SW7 2BX, United Kingdom

**Keywords:** Software verification, Software benchmark, Discrete element method, Granular mechanics

## Abstract

Since the seminal work of Cundall and Strack (1979), the Discrete Element Method (DEM) has now become accepted as a key tool amongst researchers exploring the fundamental behavior of granular materials. Along with a sustained increase in the number of publications documenting use of DEM in research, intensive development of new open-source and commercial DEM codes has taken place in the last decades. The credibility of these software packages depends on their capacity to replicate physical observations and to reproduce theoretical expressions. Researchers often calibrate DEM codes against laboratory data to gain confidence about their predictions, however, theoretical verifications at the macro and particle levels are often omitted or not explicitly documented or acknowledged. The validation of DEM codes against theoretical expressions is fundamental to guarantee reproducibility and generality of the software, and to avoid bias in more complex simulations.

In this article, a dataset providing numerical simulation data along with input files is presented. The dataset relates to a series of theoretical validation approaches, previously documented in the literature, that were here applied to verify the open-source DEM code LAMMPS. The ability of LAMMPS to capture the macroscopic behaviour of granular packages is evaluated by shearing a face-center-cubic (FCC) array of monosized spheres. The calculation of particle translational/rotational motions and forces/torques is checked by considering a clump rolling down an inclined plane. Additionally, the stress-strain behavior of Toyoura sand under “drained” and “undrained” shearing is characterized by a series of LAMMPS outputs. The dataset collected from these simulations can be employed by users to benchmark new or existing DEM codes. Both the LAMMPS input scripts and the simulation results for all the cases are available in a public repository.

Specifications Table

**Table:** 

Subject	Engineering
Specific subject area	Computational Mechanics
Data format	Raw
Type of data	Text files
Data collection	Data were generated by performing numerical simulations with the open-source software LAMMPS. Specific input scripts were generated and then ran to simulate the verification cases described in this article.
Data source location	Simulation data were generated using the Imperial College London computational facilities.
Data accessibility	Repository name: Validation and Benchmark Dataset for Discrete Element Method Simulations Data identification number: 10.5281/zenodo.10411062 (version v3) Direct URL to data: https://zenodo.org/records/10411062

## 1 Value of the Data

• The dataset contains all the macro and micromechanical information required to verify and benchmark new or existing DEM codes. Researchers may gain confidence in their predictions by first validating their code against the herein published data. The scenarios presented here follow a logical sequence that can help researchers to define a validation and benchmark procedure for their own simulations.
• The repository also provides users with input scripts that can be employed to reproduce the simulations. These inputs can be modified to densify the dataset around a specific deformation level, or they can serve as a template to simulate different testing conditions in LAMMPS.
• The cases analyzed here are all supported by a strong theoretical background. The concepts and references provided here can help users to better understand their outputs and to easily identify flaws in their procedures and code calculations.

## 2 Background

Since the seminal work of Cundall and Strack [1], the Discrete Element Method (DEM) has now become a widespread tool among researchers exploring the fundamental behavior of granular materials. Along with a sustained increase in the number of publications, intensive development of new open-source and commercial DEM codes has taken place in the last decades [2]. Software such as PFC [3], Yade [4], LIGGGHTS [5], and LAMMPS [6] have been successfully employed to model a range of granular systems including transportation and storage of powders, and mitigation of natural disasters [7].

The credibility of all these software packages depends on their capacity to replicate physical observations and to reproduce theoretical expressions [8]. Saomoto et al. [9] and Holst et al. [10] tested the predictive capability of the DEM by conducting a round-robin test on silo filling and angle of repose (AoR). Holst et al. [10] observed that the wall pressures reported by participants exhibit differences of one order of magnitude. Participants of the round robin test of AoR reported angles between 29° and 44° (mean value of 36°) for the predicted cylindrical heap measurements [9]. Both studies concluded that DEM results can be influenced by the choice of non-documented input parameters and the assumptions made in the calculations of the code. Researchers often calibrate DEM codes against laboratory data to gain confidence about their predictions, however, theoretical verifications at macro and particle level are often omitted or not explicitly documented or acknowledged. The rigorous validation of DEM codes against theoretical expressions is fundamental to guarantee the reproducibility and generality of the software, to test methodologies, and to avoid bias in more complex simulations.

In this article, a dataset providing numerical simulation data along with input files is presented. The dataset relates to a series of theoretical validation approaches, previously documented in the literature, that were here applied to verify the open-source DEM code LAMMPS. All the outputs and input scripts are included in the open repository “Validation and Benchmark Dataset for Discrete Element Method Simulations” (https://zenodo.org/records/10411062) [11]. The description of all the cases of analysis is presented in the following sections.

## 3 Data Description

**Fig. 1:**
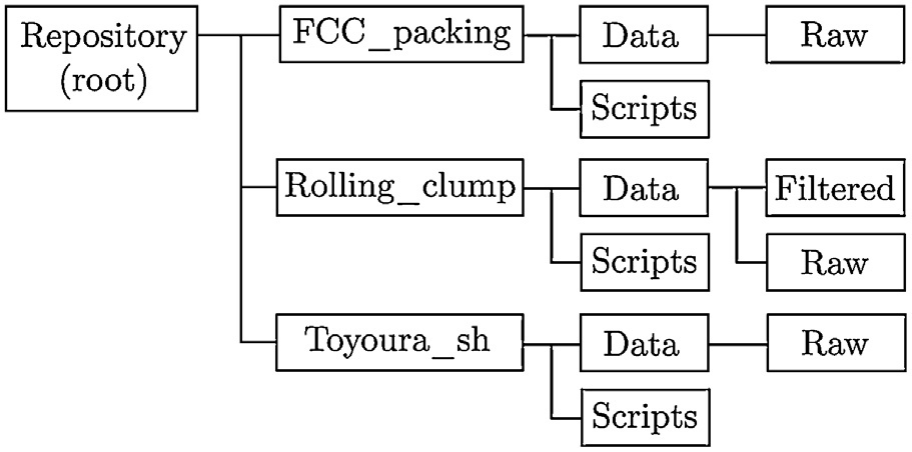
Repository structure. Name and hierarchy of the folders.

The open repository of both the verification and benchmark tests can be found at https://zenodo.org/records/10411062 [11]. The root of the repository contains a “README.txt” file that indicates the instructions to benchmark or validate existing or new DEM codes using the provided dataset. The folder structure of the repository is shown in Fig. 1. For each verification or benchmark case (namely “FCC_packing”, “Rolling_clump”, and “Toyoura_sh”), two sub-folders, called “Data” and “Scripts” can be found.

The “Scripts” folders contain all the inputs and scripts required by LAMMPS to generate the dataset herein presented. The extension of the LAMMPS script files is *.in, and the input data files (read by script files) have extensions *.lj (particle coordinates) or *.rigid (connectivity of clumps). LAMMPS can read and interpret instructions from any text file, thus the format of the input or script files is irrelevant for the software. Formats in this repository were only chosen to summarise the content or the function of the files (e.g., *.in means it is an input file).

Data contained in the “Data” folders can be raw and/or filtered (post-processed before uploading). Both “Raw” and “Filtered” sub-folders contain a README.txt file that details their file content and format. These README.txt files indicate the number of columns, the variables involved (i.e., timestep or void ratio), and labelling system employed to name the files. A chart describing the folder structure of the whole repository is shown in Fig. 1.

## 4 Experimental Design, Materials and Methods

### 4.1 Verification cases

#### 4.1.1 Rolling clump

**Fig. 2:**
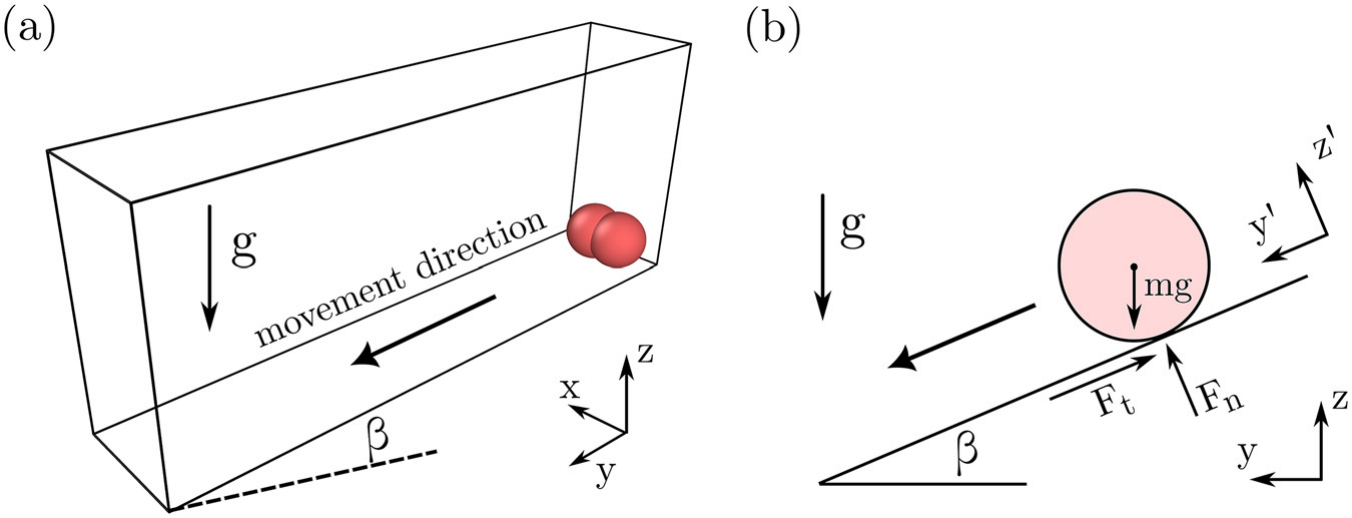
Rolling clump verification case. (a) 3D simulation setup, and (b) distribution of forces along the center of mass of the clump.

Initially proposed by Ke and Bray [12], the rolling ball test verifies the ability of the DEM to reproduce simple theoretical solutions. Normal and shear force calculations can be corroborated by this test as both types of force induce the rolling condition in particles [13]. Here, the original rolling ball test is extended to consider a 2-sphere clump particle. Clumps are rigid aggregates comprised of a set of overlapping spheres that can be employed to simulate non-spherical particle geometries. Fig. 2(a) shows the setup of the test and the movement direction of the clump particle. The clump in Fig. 2(a) is oriented such that only rotation about the x-axis is generated.

The distribution of acting forces and motions around the center of mass (CM) of the clump are illustrated in Fig.2(b). From this configuration, solutions for torque, T, and angular velocity, $\overset{˙}{\theta ,}$ can be obtained by applying rotational equilibrium about the CM of the clump, i.e.

(1) $$T=m_{clump}gcos\left(\beta \right)\tan\left(\phi \right)r;\;\overset{˙}{\theta }=\frac{m_{clump}gcos\left(\beta \right)\tan\left(\phi \right)rt}{I_{clump,xx}}$$

where β represents the plane inclination and ϕ corresponds to the friction angle, which is related to the coefficient of particle friction by $\mu =\tan^{-1}\phi$. $m_{clump}$ and $I_{clump,xx}$ correspond to the total mass and the inertia of the clump on the x-axis. r and t represent the radius of a single sphere and time, respectively.

Depending on the relation between β and ϕ, rolling or sliding conditions can take place during the test. Rolling conditions state that at the contact, angular and linear velocities, $\overset{˙}{\theta }$ and $v_{y}$, must coincide. Mathematically this means:

(2) $$\overset{˙}{\theta }r=-v_{y};\;v_{y}=g\left(\text{cos}\left(\beta \right)\tan\left(\phi \right)-\text{sin}\left(\beta \right)\right)t$$

From where a limit value for the angle of friction, $\phi_{lim}$, can be established. If $\phi \geq \;\phi_{lim}$ the particle rolls, otherwise, only sliding occurs. The limit angle of friction is given by,

(3) $$\;\phi_{lim}=tan^{-1}\left(\frac{\text{tan}\left(\beta \right)}{1+\frac{m_{clump}r^{2}}{I_{clump,xx}}}\right)$$

Using $\phi_{lim}$, the analytical solutions for torque, T, and angular velocity, $\overset{˙}{\theta }$, from Eq. (3) can be set to meet rolling conditions, i.e,

(4) $$T=\frac{m_{clump}g\text{sin}\left(\beta \right)r}{1+\frac{m_{clump}r^{2}}{I_{clump,xx}}};\;\overset{˙}{\theta }=\frac{m_{clump}g\text{sin}\left(\beta \right)}{I_{clump,xx}+m_{clump}r^{2}}t$$

In this article, Eq. (3) and Eq. (4) are compared against LAMMPS simulations of a rolling clump. Different angles of friction and a single plane inclination are considered for this purpose. Details about the input parameters and configuration can be found in the “DATA GENERATION” section.

#### 4.1.2 FCC packing

**Fig. 3:**
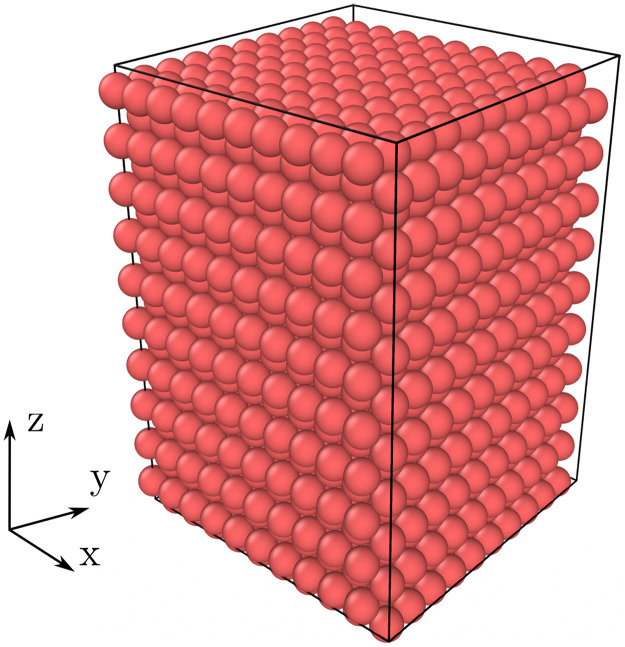
Face-centered-cubic (FCC) packing verification case. Simulation setup.

The ability of LAMMPS to capture the macroscopic behavior of granular packages is evaluated by shearing in traxial compression a face-center-cubic (FCC) array of monosized spheres (Fig. 3). This verification case has been included in PFC documentation alongside releases of the software [3].

Thornton [14] derived a solution for peak stress or failure conditions considering a periodic space in all directions, no particle rotation during shearing, and equal directions between the stress and strain incremental tensors. The peak stress ratios, $\sigma_{ii}/\sigma_{jj}\;$for i,j∈{x,y,z}, under triaxial loading can be expressed by:

(5) $$\frac{\sigma_{zz}}{\sigma_{xx}}=\;\frac{2+2\mu /F}{1-2a\mu /F};\;\frac{\sigma_{yy}}{\sigma_{xx}}=\;\frac{1-2b\mu /F}{1-2a\mu /F};\;F=\sqrt{\frac{3}{2}\left(a^{2}+b^{2}\right)+ab}$$

Where aand b represent two constants that set the relationship between the intermediate and minor principal stresses ($\sigma_{xx}$ and $\sigma_{yy}$). This solution considers that the array is loaded in the z-direction.

It is important to note that the solution given by Eq. (5) is independent of the interparticle contact model and the input parameters (e.g., shear modulus, damping ratio, and density). Eq. (5) depends only on the interparticle friction μ and the loading conditions (aand b), thus different contact models or inputs must deliver identical stress ratios at failure.

Here the solution given by Eq. (5) is compared against LAMMPS simulations of an FCC array of spheres. A single interparticle friction value is tested and included in the repository. Details about the input parameters and configuration can be found in the “DATA GENERATION” section.

### 4.2 Benchmark tests

In addition to the two verification cases, the repository includes a benchmark dataset for Toyoura sand. In this dataset, the “drained” and “undrained” stress-strain behavior of a polydisperse sample of 20,174 spheres is characterized by a series of LAMMPS outputs. Particle radii were selected to resemble the particle size distribution (PSD) from Yang & Sze [17]. “Drained” shearing conditions mean that lateral stresses are kept constant and “undrained” means that the volume is kept constant during the simulations. Both types of shearing are common and relevant testing conditions in geotechnical practice.

Using similar simulation conditions and particle characteristics, Huang et al. [15] demonstrated that a qualitative agreement between the DEM data and the experimental database of Jefferies and Been [16] can be achieved. The dataset herein collected can be employed by users to benchmark new or existing DEM codes. Details about the input parameters and configuration can be found in the “DATA GENERATION” section

### 4.3 Data generation

The dataset herein presented was entirely generated by LAMMPS scripts. The input and script files for the different cases can be found in their respective “Scripts” sub-folders. The *.in files are scripts that contain a series of instructions and commands that are interpreted by LAMMPS to compute calculations. The initial position of the particles is specified in the *.lj files, which can also be used as input in different DEM software (e.g. Yade, PFC). Details on how to run and reproduce the repository data can be found in the “README.txt” file contained in every “Scripts” folder. In order to reproduce the simulations of this repository, LAMMPS must be built including the “GRANULAR” and “RIGID” packages. All the LAMMPS input scripts for the validation cases have been successfully tested using the git version published on 3/03/2020. All the LAMMPS input scripts for the benchmark tests have been successfully tested using the git version published on 28/03/2023.

Users aiming to reproduce the rolling clump verification test must run the “test_clumps_plane_Test_clump1.in” (sphere) or the “test_clumps_plane_Test_clump15.in” (clump) script. In the simulations, the inclination of the plane is controlled by decomposing gravity in two directions. Gravity is applied in LAMMPS by using the “fix gravity” command. A summary of the simulation parameters can be found in Table 1.

**Table 1:** Simulation parameters. Rolling clump verification case.

Parameter	Value	Unit
Contact model	Hertz-Mindlin	
Plane inclination	45	^o^
Particle Radius	5 × 10^−1^	m
Young Modulus,E	60	GPa
Poison's ratio, ν	0.2	-
Particle density, ρ	2,650	kg/m^3^
Angle of friction, ϕ	5,10,15,20,30,40, 60	^o^
Timestep, Δt	1 × 10^−5^	s
Damping coefficient (Tsuji), e	0.05	-

The FCC packing verification dataset can be replicated by sequentially running the “Isotropic_mu0_0.0.in”, “Isotropic_eq_mu0_0.0_muf_0.5.in”, and “Shearing.in” script files. During the simulations, the lateral stresses are kept constant (constant $\sigma_{3}$, a=b=0.5) by introducing a Berendsen-type barostat through the “fix press/berendsen” command. This barostat is coupled to the shearing deformation in the z-direction that is applied by using the “fix deform” command. A summary of the simulation parameters can be found in Table 2.

**Table 2:** Simulation parameters. FCC packing verification case.

Parameter	Value	Unit
Contact model	Hertz-Mindlin	
Number of particles	2000	-
Particle Radius	1 × 10^−2^	m
Shearing rate, $v_{z}$	0.001	m/s
Shear modulus, G	29	GPa
Poison's ratio, ν	0.12	-
Particle density, ρ	2,670	kg/m^3^
Coefficient of friction, μ	0 (Isotropic compression) 0.5 (Shearing)	-
Timestep, Δt	1 × 10^−7^	s
Local damping, η	0.1	-
Effective confining pressure, ${\sigma_{c}}^{\prime }$	100	kPa

To reproduce the benchmark dataset for Toyoura sand, users must sequentially run the “Isotropic_mu0_0.13.in”, “Isotropic_eq_mu0_0.13_muf_0.25.in”, and “Shearing_DR.in” or “Shearing_UN.in” script files. During “drained” shearing, lateral stresses are kept constant following the procedure described for the FCC packing. The “undrained” condition is achieved by applying constraints to the deformation of the sample through the “fix deform volume” command. A summary of the simulation parameters can be found in Table 3.

**Table 3:** Simulation parameters. Toyoura sand benchmark simulations.

Parameter	Value	Unit
Contact model	Hertz-Mindlin	
Number of particles	20,172	-
Shearing rate, $v_{z}$	0.001	m/s
Shear modulus, G	29	GPa
Poison's ratio, ν	0.12	-
Particle density, ρ	2670	kg/m^3^
Coefficient of friction, μ	0.13 (Isotropic compression) 0.25 (Shearing)	-
Timestep, Δt	5.37 × 10^−6^	S
Local damping, η	0.1	-
Effective confining pressure, ${\sigma_{c}}^{\prime }$	300	kPa

**Fig. 4:**
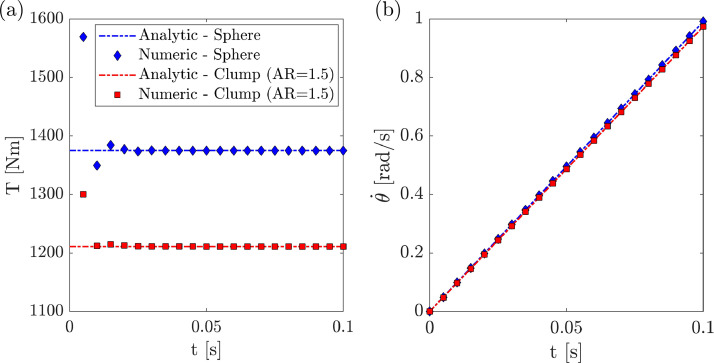
Rolling clump verification case. Simulation dataset and analytical solution. (a) Torque v/s time and (b) angular velocity v/s time.

**Fig. 5:**
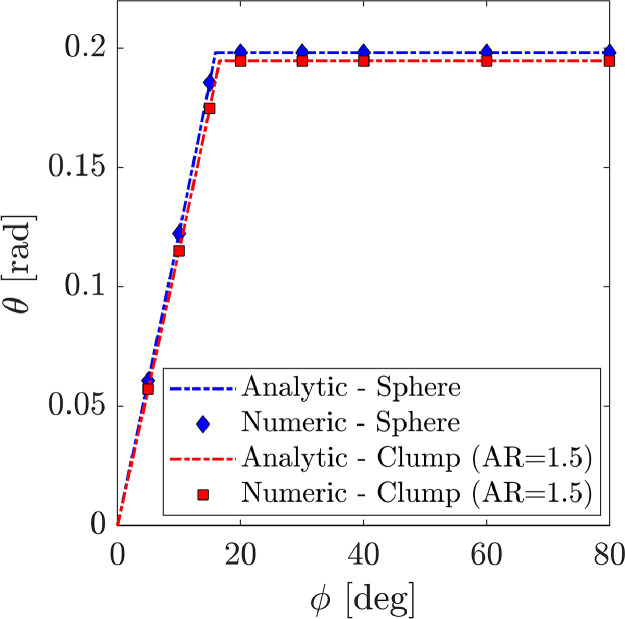
Rolling clump verification case. Simulation dataset and analytical solution. Angular displacement v/s coefficient of interparticle friction.

Figs. 4 and 5 display the verification dataset for the rolling clump tests. Fig. 4 compares the analytical solution (Eq. (4)) and the output of the simulations in terms of the torque, T, and the angular velocity, $\overset{˙}{\theta ,}\;$in the contact plane. Results shown here were obtained for an inclination of$\;\beta =45^{o}$ and an angle of interparticle friction of $\phi =60^{o}$ only. A unique aspect ratio of AR=1.5was considered to assemble the clump particle. The aspect ratio, AR here is considered as the ratio between the shortest and longest dimension of the clump. A full description of the parameters employed in the simulations can be found in the next section.

Fig. 5 shows the terminal torque, Tand the particle angular rotation, θfor both the simulation results and the analytical solution (Eq. (3)). Values shown in the plot were obtained by post-processing the dump.* files included in the “Raw” folder. The MATLAB file “rolling_clump_plots.m”, found in the “Filtered” folder, can be used to reproduce the plots in Figs. 4 and 5.

**Fig. 6:**
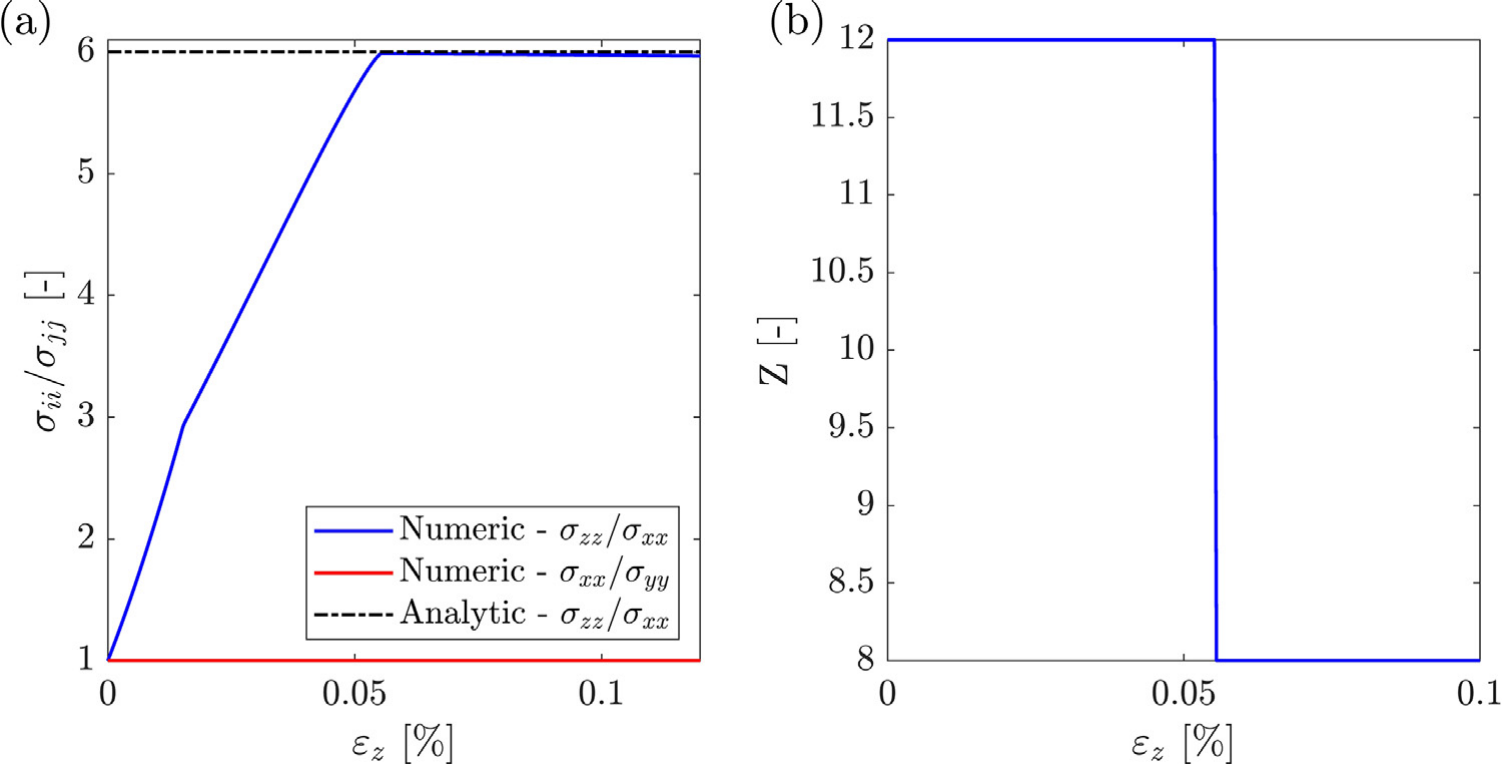
FCC packing verification case. Shearing application. (a) stress ratio v/s axial strain, and (b) coordination number v/s axial strain.

The verification dataset for the FCC packing is graphically presented in Fig. 6. Fig. 6(a) shows the simulation results and the analytical solution (Eq. (5)) in terms of the stress ratio, $\sigma_{ii}/\sigma_{jj}$ and the axial strain, $\varepsilon_{z}$. Fig. 6(b) exhibits the variation of the coordination number, Z, defined as the average number of contacts per particle. A coefficient of interparticle friction of μ=0.5 and a loading condition of a=b=0.5was considered in all the simulations. A stress ratio of $\sigma_{zz}/\sigma_{xx}$=6 and a coordination number of Z=12 are predicted by the analytical solution. The MATLAB file “plot_FCC.m”, found in the “Raw” folder, can be used to reproduce Fig. 6.

**Fig. 7:**
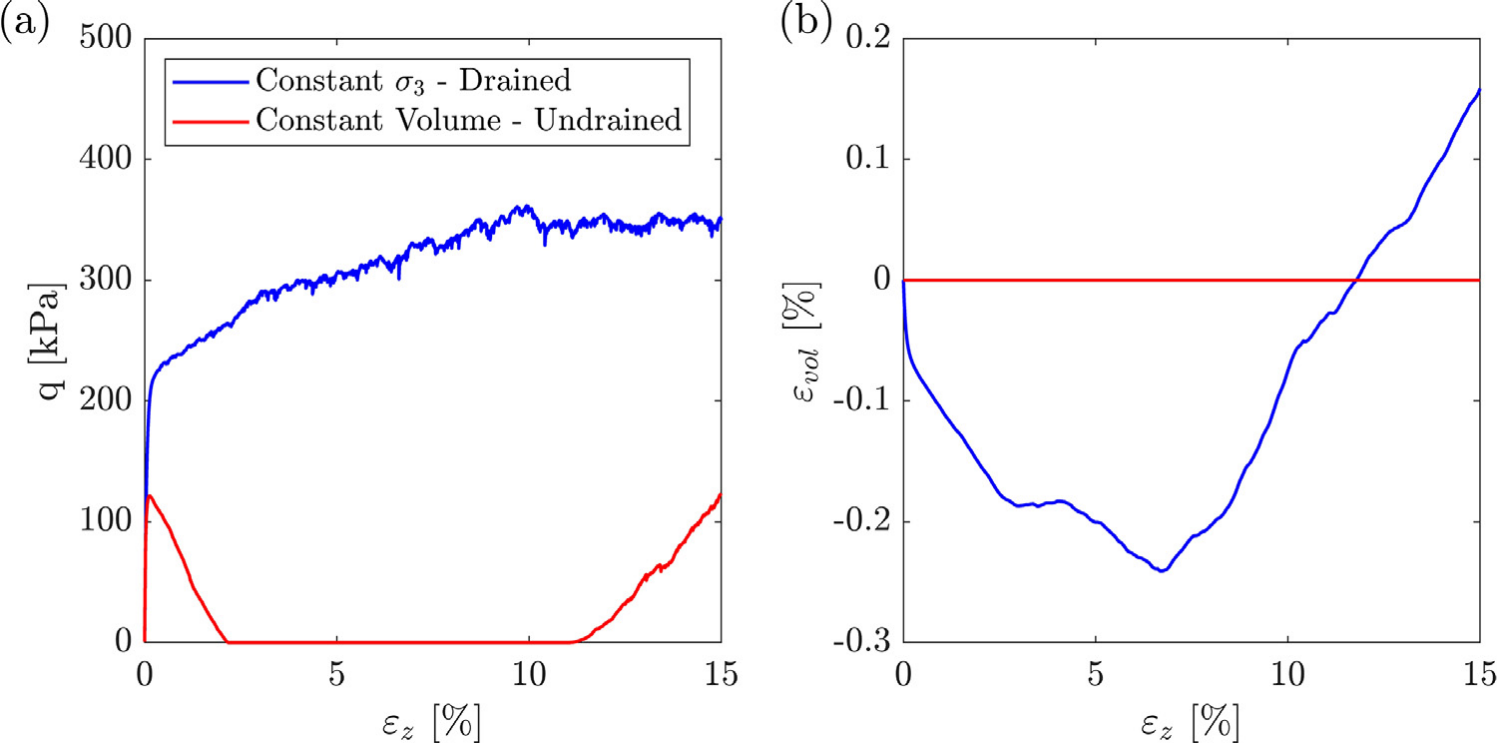
Toyoura sand benchmark dataset. “Drained” and “undrained” triaxial shearing. (a) deviatoric stress v/s axial strain, and (b) volumetric strain v/s axial strain.

The benchmark dataset for Toyoura sand can be observed in Fig. 7. Fig. 7(a) shows the change in the deviatoric stress $q=\sigma_{1}-\sigma_{3}$ with axial strain, $\varepsilon_{z}$. Fig. 7(b) displays the variation of the volumetric strain, $\varepsilon_{vol}$ as a function of $\varepsilon_{z}$. “Drained” and “undrained” simulations are shown in blue and red, respectively. The MATLAB file “plot_toyoura.m”, found in the “Raw” folder, can be used to reproduce Fig. 7.

## Limitations

None.

## Ethics Statement

The authors agree with the ethical requirements for publication in Data in Brief and confirm that the current work does not involve human subjects, animal experiments, or data collected from social media.

## CRediT authorship contribution statement

**Jose Salomon:** Conceptualization, Methodology, Software, Validation, Data curation, Writing – original draft, Visualization. **Catherine O'Sullivan:** Conceptualization, Methodology, Writing – review & editing. **Fernando Patino-Ramirez:** Conceptualization, Methodology, Writing – review & editing.

## Data Availability

Validation and Benchmark Dataset for Discrete Element Method Simulations (Original data) (Zenodo).
